# Assessing pandemic preparedness, response, and lessons learned from the COVID-19 pandemic in four south American countries: agenda for the future

**DOI:** 10.3389/fpubh.2023.1274737

**Published:** 2023-11-29

**Authors:** Andrea Ramírez Varela, Michael Touchton, J. Jaime Miranda, Juliana Mejía Grueso, Rachid Laajaj, Gabriel Carrasquilla, Martha Vives Florez, Ana María Vesga Gaviria, Ana María Ortiz Hoyos, Esteban Orlando Vanegas Duarte, Alejandra Velásquez Morales, Nubia Velasco, Silvia Restrepo Restrepo

**Affiliations:** ^1^School of Medicine, Universidad de los Andes, Bogotá, Colombia; ^2^Faculty Lead for Global Health, Institute for Advanced Study of the Americas, University of Miami, Coral Gables, FL, United States; ^3^School of Medicine, Universidad Peruana Cayetano Heredia, Lima, Peru; ^4^Department of Economics, Universidad de los Andes, Bogotá, Colombia; ^5^Academia Nacional de Medicina, Bogotá, Colombia; ^6^Department of Biological Sciences, Universidad de los Andes, Bogotá, Colombia; ^7^Asociación Nacional de Empresarios, Bogotá, Colombia; ^8^Fundación Santo Domingo, Bogotá, Colombia; ^9^Health Sciences School of Medicine, Universidad El Bosque, Bogotá, Colombia; ^10^School of Business, Universidad de los Andes, Bogotá, Colombia; ^11^Department of Food and Chemical Engineering, Universidad de los Andes, Bogotá, Colombia

**Keywords:** COVID-19, preparedness and response, lessons learned, COVID-19 pandemic, South America

## Abstract

**Introduction:**

The COVID-19 pandemic emerged in a context that lacked adequate prevention, preparedness, and response (PPR) activities, and global, regional, and national leadership. South American countries were among world’s hardest hit by the pandemic, accounting for 10.1% of total cases and 20.1% of global deaths.

**Methods:**

This study explores how pandemic PPR were affected by political, socioeconomic, and health system contexts as well as how PPR may have shaped pandemic outcomes in Argentina, Brazil, Colombia, and Peru. We then identify lessons learned and advance an agenda for improving PPR capacity at regional and national levels. We do this through a mixed-methods sequential explanatory study in four South American countries based on structured interviews and focus groups with elite policy makers.

**Results:**

The results of our study demonstrate that structural and contextual barriers limited PPR activities at political, social, and economic levels in each country, as well as through the structure of the health care system. Respondents believe that top-level government officials had insufficient political will for prioritizing pandemic PPR and post-COVID-19 recovery programs within their countries’ health agendas.

**Discussion:**

We recommend a regional COVID-19 task force, post-pandemic recovery, social and economic protection for vulnerable groups, improved primary health care and surveillance systems, risk communication strategies, and community engagement to place pandemic PPR on Argentina, Brazil, Colombia, and Peru and other South American countries’ national public health agendas.

## 1 Introduction

On January 30, 2020, the World Health Organization (WHO) declared that the SARS CoV-2 outbreak, which causes the COVID-19 disease, was a global health emergency, and on March 11, 2020, it was declared a pandemic (1). As of July 2023, there were over 767 million cases reported globally, with 35.9% in Europe (EURO), 26.6% in the Western Pacific (WPRO), 25.2% in the Americas (PAHO), 8.0% in South-East Asia (SEARO), 3.0% in the Eastern Mediterranean (EMRO), and 1.2% in Africa (AFRO). There were 6.9 million confirmed global deaths from COVID-19 (1), of which 42.6% were in PAHO, 32.3% in EURO, 11.6% in SEARO, 6.0% WPRO, 5.1% in EMRO, and 2.5% in AFRO. Despite having just 5.5% of the world’s population, South American countries (comprising Argentina, Bolivia, Brazil, Chile, Colombia, Ecuador, Paraguay, Peru, Uruguay, and Venezuela, according to the Pan American Health Organization [PAHO] list (2)) were some of the hardest hit, accounting for 10.1% of total cases and 20.1% of global deaths (3). As of June 2023, Brazil had 57.5% of South America’s cases (equivalent to 17.6% of its population), followed by Argentina (15.3%, equivalent to 22.2% of its population), Colombia (9.7%, equivalent to 12.4% of its population), and Peru (6.9%, equivalent to 13.4% of its population) (3). Of these four countries, Peru had the highest case-fatality rate (4.9%) and Argentina had the lowest (1.3%) (3).

According to the 2021 Global Health Security (GHS) Index, possible explanations for South America’s poor health outcomes during the COVID-19 pandemic include: (a) pre-pandemic conditions (e.g., high population density, health inequalities, high informal employment, lack of Universal Health Coverage [UHC], and poverty); (b) the high burden of disease due to “the silent pandemic” of noncommunicable diseases (NCDs); (c) the lack of preparedness for epidemic and pandemic responses; (d) a complex political climate in which corruption, among other factors, weakened trust in public guidance, public policy, and health interventions; (e) weak governance that prevented COVID-19 reaction evidence-based decision-making; and (f) weak health services (e.g., primary health care [PHC]). The index uses 37 indicators to assess a country’s capacity for prevention, detection, and response to biological threats, and health systems, norms, and risks that impede or improve that preparedness (4). South American countries had scores between 20.9 and 56.2 on a scale of 0 to 100, which are among the lowest when compared to North American and European countries (5).

Yet, there are currently no systematic studies of policy experts’ opinions in South America at the national and regional levels about the most pressing issues to be addressed three years into the pandemic and the form that future responses and preparedness for health emergencies should take. To achieve an effective response to future global health emergencies, it is fundamental to assess the determinants of pandemic prevention, preparedness, and response activities. In turn, understanding these processes allows for a synthesis of lessons from the current pandemic and a consolidation of available evidence for decision making and governance to address the regions’ most pressing public health issues. Placing decision-makers’ opinions in the context of national COVID-19 responses, and, by extension public policymaking in general thus provides a foundational step toward a nuanced policy framework for public health.

The authors’ conceptual framework is based on the political economy of health, eco-social models of health (6), and the policy response framework from the University of Miami’s Observatory for the Containment of COVID-19 (7). Using these frameworks contributes to the understanding and characterization of the distinct challenges that South American countries such as Argentina, Brazil, Colombia, and Peru faced during the COVID-19 pandemic and, why they responded differently through public policy and non-pharmaceutical interventions (NPI) in 2020 and 2021.

Under these frameworks, the authors use key informant interviews to evaluate the political, economic, social, and environmental factors that may have influenced COVID-19 PPR activities (8), and their possible correlation with health outcomes (e.g., COVID-19 incidence, deaths, and vaccination rates).

Therefore, this study explores the extent to which political, socioeconomic, and health system contexts affected the COVID-19 pandemic prevention, preparedness, and response (PPR) during 2020–2021 across four South American countries: Argentina, Brazil, Colombia, and Peru. This study also explores how political, socioeconomic, and health system contexts may have shaped COVID-19 incidence, deaths, and vaccine coverage in these countries drawing from decision makers’ perspectives, and identifies lessons learned and institutional priorities for future pandemic PPR. In doing so, this study provides evidence for policy recommendations and an agenda for improving regional and national capacity for future pandemics.

## 2 Materials and methods

### 2.1 Study design

This is a mixed-methods sequential explanatory study, combining collection and analyses of quantitative and qualitative data (9).

### 2.2 Setting

This study included the four South American countries with the largest populations and the most confirmed cases and deaths due to COVID-19: Argentina, Brazil, Colombia, and Peru (1–3). This study was conducted through a research collaboration in 2022 between Universidad de los Andes, Colombia, the Open Society University Network OSUN, and the Queen Elizabeth II Academy for Leadership in International Affairs, Chatham House, United Kingdom.

#### 2.2.1 Instrument development

**Figure 1:**
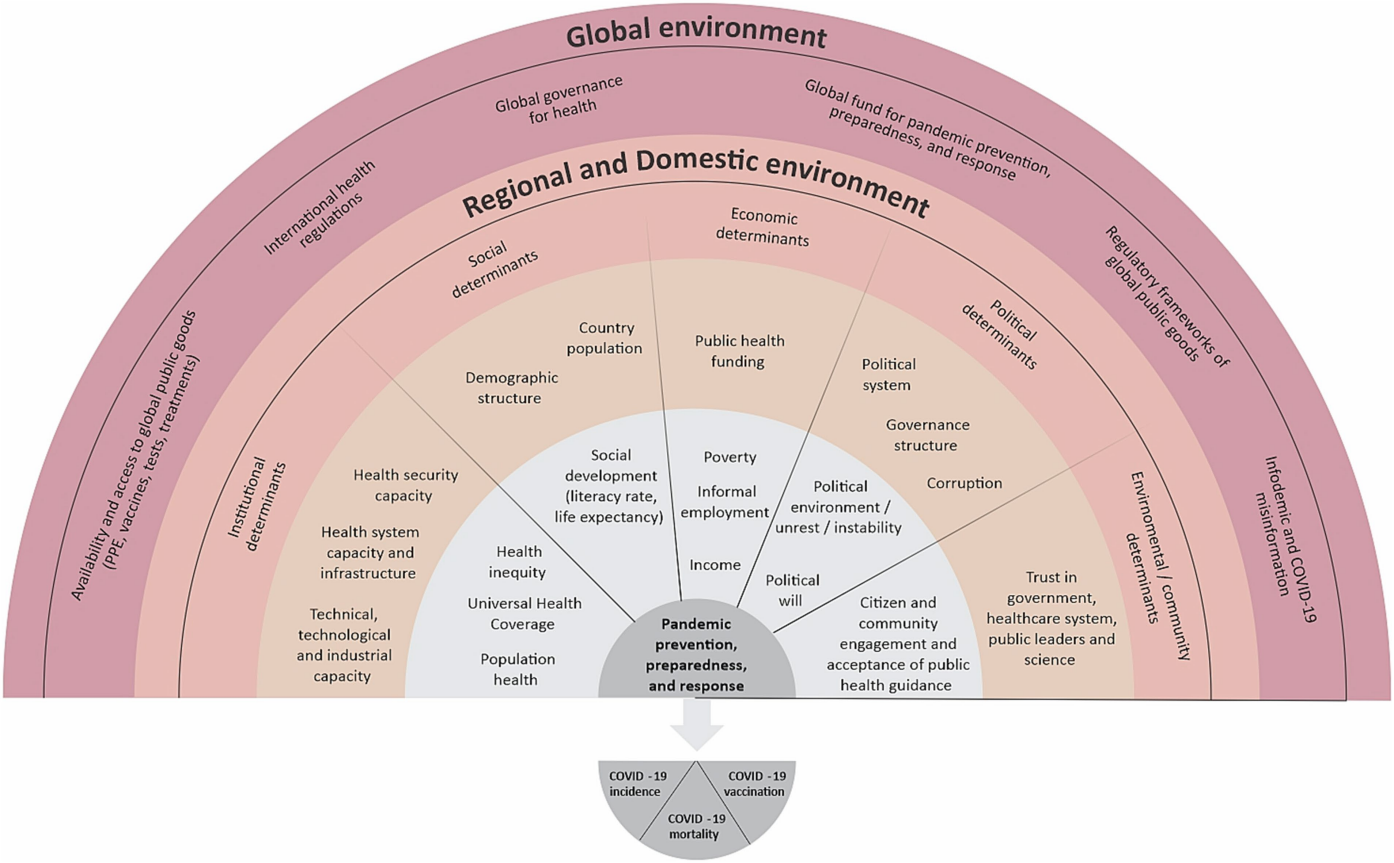
Multidimensional and Multilevel Conceptual Framework for the COVID-19 Pandemic Prevention, Preparedness, and Response (PPR). Based on frameworks from Huynen et al. (6), Bollyky et al. (8), and the University of Miami’s Observatory for the Containment of COVID-19 (7).

A country profile was built using secondary data sources to collect the most recent data on the institutional, social, economic, political, and environmental / community indicators, as shown in Figure 1. The WHO (10), PAHO (11), World Bank (12), Inter-American Development Bank (13), Our World in Data (3), United Nations (UN) (14), Economic Commission for Latin America and the Caribbean (15), public databases, and other documents provided data for these indicators. This profile was included in the first part of the survey questionnaire to facilitate instrument application and provide participants with their country’s context.

The instrument was developed based on the UN and WHO’s preparedness definition (16), the WHO COVID-19 Strategic Preparedness and Response Plan (17), and the GHS Index (4). Also, taking into account the GHS Agenda targets of prevention, detection, and response to combat infectious diseases and build a resilient public health system (18):

- Prevention activities:

a) Attention to zoonotic diseases reported in surveillance systems.
b) Bioprotection and biosecurity measures, systems, training activities to ensure protection and safety when handling biological material.
c) Regular immunization programs.

- Detection and reporting activities:

a) The existence of a national laboratory system with the capacity to detect prioritized diseases, referral, and transport of samples.
b) Detection and notification of events of public health interest with surveillance systems for real-time detection.
c) Accessibility and transparency of epidemiological surveillance data.
d) Real-time surveillance systems’ reports, data accessibility and transparency.

- Response activities:

a) Emergency operations centers and/or early warning systems for the detection of public health events.
b) Multisectoral response and risk communication.
c) Medical countermeasures and health personnel deployment.
d) Installed capacity, supply chain, and medical care in clinics, hospitals, care centers.

The sections of the instrument were as follows:

- Assessments of pandemic PPR plans and perceptions of relations between prevention, detection, reporting, and response activities (7) and COVID-19 health outcomes (total cases, deaths, and vaccination rates). Responses were coded on a four-point Likert scale that categorized elements of PPR as highly correlated, somewhat correlated, somewhat uncorrelated, or highly uncorrelated with health outcomes.
- Assessments of connections between the variables in Figure 1 and pandemic PPR activities. Responses were coded on a four-point Likert scale categorizing variables as factors that facilitated, somewhat facilitated, somewhat hindered, or hindered PPR.
- Open-ended questions to identify the top facilitators, top factors that were hardest to manage, and lessons learned from the pandemic PPR process.
- Assessments of decision makers’ PPR support, health authorities and policymakers’ priorities for the next year, and the most significant organizations and institutions in the country. Responses were coded on a four-point Likert scale evaluating decision-makers as very supportive, somewhat supportive, not very supportive, or not at all supportive.
- There were two versions of the questionnaire used in surveys (see Supplementary material S1, S2): a 46-question version shared with country representatives to collect country data and a five-question version shared with participants from multilateral organizations working in the region and not only in one country. Five academics (holding a university position), first responders (paramedics, emergency medical technicians), and field epidemiologists were interviewed from February to March 2022 to pilot test the instrument’s applicability, comprehensiveness, acceptability, and practicality.

### 2.3 Participants

Through a purposive sampling strategy, we aimed to include key informants at the highest level of decision making that were also directly involved in their country’s health policy responses during the first two years of the COVID-19 pandemic.

The list of candidates for participation was carefully developed through a collaboration between the lead author, experts from the Centre for Universal Health and the Latin America, U.S., and the Americas Programme at Chatham House / The Royal Institute of International Affairs, United Kingdom, and public health experts from the CoVIDA project engaged in the COVID-19 response in Colombia at Universidad de los Andes, in Bogotá, Colombia (19). The list of potential participants included at least one representative per country in each of three areas (1) national and subnational governments, (2) academia and civil society, and (3) multilateral organizations.

As a result, the list of potential participants included 65 key informants working during the first two years of the pandemic as: (1) national health ministers; (2) secretaries of health at the subnational level; (3) lead members of national institutes of health and epidemic surveillance officers; (4) presidents of national and regional emergency medicine societies; (5) academics (holding a university position) leading key research epidemiologic surveillance, pandemic response, clinical trials and/or COVID-19 vaccination studies for the country; (6) private entrepreneurs and philanthropists funding multiple initiatives to mitigate the pandemic; and (7) high level representatives from multilateral organizations such as the Pan American Health Organization PAHO, Inter-American Development Bank IBD, United Nations Development Programme UNDP, Organismo Andino de Salud CONHU, National Association of Entrepreneurs of Colombia ANDI, among others in the region.

Therefore, the key informants in this study were among the most knowledgeable actors in these countries on pandemic preparedness and response during the first two years of the COVID-19 pandemic. This multidisciplinary group included experts from the following areas: epidemiology, public health, medical sciences, social sciences, political sciences, law, economy, philanthropy, and management. The key informants were publicly known actors in each of their countries and their involvement in the COVID-19 pandemic response was thoroughly described and recorded in public documents, social media, and institutional websites.

### 2.4 Conceptual framework

The authors developed a conceptual framework based on the political economy of health, eco-social model of health (6), and the policy response framework from the University of Miami’s Observatory for the Containment of COVID-19 (7) conceptual frameworks. The resulting conceptual framework included the political, economic, social, and environmental factors that may have influenced the COVID-19 PPR activities (8), and possible health outcomes (e.g., COVID-19 incidence, deaths, and vaccination rates). Figure 1 provides a graphical depiction of this framework showing the vertical and horizontal factors influencing pandemic PPR in a given context.

### 2.5 Data collection

#### 2.5.1 Interviews

Data collection was conducted from April 26 to May 13, 2022, through structured interviews with key informants using an online instrument. Consent was obtained prior to the interviews and participants were emailed the set of closed- and open-ended questions. The interviews were undertaken by the lead author using a structured interview guide, lasting an average of 45 min for the long version and 25 min for the short version (see section 2.2. Instrument Development for details). All interviews were recorded using Zoom and were transcribed verbatim. Participants were required to speak English, Spanish, or Portuguese.

#### 2.5.2 Expert focus groups

Two hybrid expert focus groups with in-person and online participants were held to complement the interviews by synthesizing regional pandemic PPR lessons and proposing recommendations for a future regional agenda. These conversations also led to recommendations to reduce regional health impacts, improve population health, and end the COVID-19 pandemic. Interviewees and other important stakeholders participated in expert focus groups in London and Bogotá in May 16 and November 2, 2022, respectively.

### 2.6 Data analysis

#### 2.6.1 Methods for analyzing quantitative data

The survey was password protected, and only accessible by the study’s researchers. Descriptive analyses were conducted using Stata (version 17.0, StataCorp, College Station, TX, US) to identify associations between pandemic PPR activities and health outcomes, barriers to and facilitators of pandemic PPR actions, and levels of support for PPR actions. The graphs were developed in R (version 4.1.3, R Foundation for Statistical Computing, Vienna, Austria).

#### 2.6.2 Methods for analyzing qualitative data

The open-ended questions in the survey were designed to elicit responses about the pandemic PPR process’s strengths, weaknesses, and lessons learned in each country or region. The MAXQDA (VERBI Software 2021) qualitative data analysis tool and codebook were used to evaluate transcripts.

##### 2.6.2.1 Data coding

For transcript analysis, a codebook was created with anticipated themes. Deductive-based coding was utilized to better convey underlying ideas in interview transcripts. Two major groups and nine themes emerged based on interview transcripts’ most common subjects and guidance from Figure 1.

###### 2.6.2.1.1 Regional themes

1. Health sovereignty: including countries facing the pandemic as a bloc to ensure global public health goods (e.g., personal protective equipment, supplies, vaccines), local scientific studies, and research capacity.
2. Politics and politicization of the COVID-19 pandemic: including corruption, science denialism, political environments, electoral periods, and their influence in PPR implementation or mis-implementation (20).
3. Multisectoral action and collaboration: including pandemic control and mitigation efforts.

###### 2.6.2.1.2 Country-specific themes

1. Countries’ political, social, and economic context: including social and health inequities, trust between the government, public institutions, scientists, public leaders, and citizens, social protection activities, poverty, and job informality.
2. Pandemic governance: including mis- and disinformation, effective risk communication, transparency in public health decision-making, sharing experiences with other countries, scientific data availability and evidence-based decision-making, translational research, and visibility of public health importance.
3. Implementation of PPR plans: including policy availability, vaccination program experience, infectious disease management and nonpharmacological approaches, community leadership and involvement, political will, competency, and leadership.
4. Pandemic funding: including state funding for PPR activities and for addressing post-COVID-19 health consequences.
5. National public health / epidemic surveillance system: including health security legislation and activities, and epidemiologic and genomic surveillance capacity.
6. National Health System: including structure, operations, resources, resilience, adaptation, UHC, PHC, and post-COVID-19 recovery.

### 2.7 Ethical approval

Ethics approval was obtained from the Ethics Committee of Universidad de los Andes (2022; Approval No. 20220401).

## 3 Results

The study included representatives from Argentina, Brazil, Colombia, and Peru. Chile was excluded due to unresponsive contacts. From the list of 65 key informants that were invited to participate in the study, 24 (36.9%) had incorrect email addresses, 12 (18.4%) declined the invitation due to unavailability during that time period, and 29 (44.6%) accepted the invitation. From these, 24 (82.8%) interviews were scheduled and completed, and after multiple attempts five (17.2%) were not scheduled due to conflicting schedules. Argentina, Colombia, and Peru featured representation from at least one member of national/subnational governments, academia, or a multilateral organization. For Brazil, relevant government representatives were contacted without reply. Therefore, the distribution of participants according to their background was nine (37.5%) government officials at national / subnational level, nine (37.5%) from multilateral organizations, and six (25.0%) academics. Key respondents represented 19 organizations in four countries and five multilateral organizations in South America. Sixteen participants completed the long version of the instrument (66.7%), while eight completed the shorter version (33.3%).

More than 80% of the participants perceived that the GHS Agenda’s aims of prevention, detection, and response to combat infectious disease risks and develop a resilient public health system (21) were positively related with total COVID-19 cases in each country. The detection and notification of events of public health interest with surveillance systems for real-time detection (87.5%), accessibility and transparency of epidemiological surveillance data (81.3%), and the existence of a national laboratory system with capacity and quality for priority disease detection, referral, and sample transport (81.3%) were the most common factors influencing COVID-19 prevention efforts (see Table 1, and for quantitative results see Supplementary material S3–S5). In addition, accessibility, and transparency of epidemiological surveillance data (68.8%); installed capacity, supply chain, and medical care in clinics, hospitals, care centers (68.8%); and multisectoral response and risk communication (68.8%) were perceived as related with COVID-19 deaths in each country. Also, multisectoral response and risk communication (68.8%), accessibility and transparency of epidemiological surveillance data (62.5%), and regular immunization programs (62.5%) were perceived as related with COVID-19 vaccinations.

**Table 1:** Main pandemic prevention, preparedness, and response items perceived as related with COVID-19 incidence, deaths, and vaccination rates*.

Pandemic prevention, preparedness, and response items	COVID-19 incidence					COVID-19 deaths					COVID-19 vaccination				
	Overall countries	Argentina	Brazil	Colombia	Peru	Overall countries	Argentina	Brazil	Colombia	Peru	Overall countries	Argentina	Brazil	Colombia	Peru
Accessibility and transparency of epidemiological surveillance data	✓	✓	✓		✓	✓	✓	✓		✓	✓	✓	✓		
Attention to zoonotic diseases reported in surveillance systems					✓										
Bioprotection and biosecurity measures, systems, training activities to ensure protection and safety to handle biological material				✓						✓					
Detection and notification of events of public health interest with surveillance systems for real-time detection	✓	✓		✓											
Existence of a national laboratory system with capacity and quality for the detection of priority diseases, referral and transport of samples	✓				✓										
Installed capacity, supply chain and medical care in clinics, hospitals, care centers						✓			✓						
Medical countermeasure and health personnel deployment		✓				✓			✓		✓				✓
Multisectoral response and risk communication			✓			✓	✓	✓		✓	✓	✓	✓	✓	✓
Real time surveillance systems’ report, data accessibility and transparency			✓	✓				✓	✓					✓	
Regular immunization programs											✓		✓	✓	✓

*Long version of the instrument, *n* = 16 interviewees.

### 3.1 Implementation of pandemic PPR

Public trust in science—in scientists and scientific evidence—most facilitated PPR implementation. Yet, NCDs, the leading cause of death in each country, hindered pandemic PPR activities (see Table 2, Experts’ Perceptions of Pandemic PPR by Thematic Category section, and Supplementary material S6 for quantitative results).

**Table 2:** Main factors perceived as facilitators or barriers for pandemic prevention, preparedness, and response activities during the first two years of the COVID-19 Pandemic.*.

Factors that facilitated/somewhat facilitated or hindered/somewhat hindered pandemic prevention, preparedness, and response activities	Overall countries	Argentina	Brazil	Colombia	Peru
*Facilitated/somewhat facilitated*					
Trust in science	✓	✓	✓	✓	✓
Social development	✓	✓		✓	✓
Universal Health Coverage	✓		✓	✓	✓
Health security capacity	✓		✓	✓	
Public health funding	✓	✓		✓	
Health system capacity and infrastructure		✓	✓		
Demographic structure		✓			✓
Citizen and community engagement and acceptance of public health guidance					✓
Trust in the healthcare system			✓		
*Hindered/somewhat hindered*					
Population health (burden due to NCDs)	✓	✓	✓	✓	✓
Corruption	✓	✓	✓	✓	✓
Infodemic and COVID-19 misinformation	✓	✓			
Trust in public leaders	✓	✓			
Poverty	✓		✓	✓	✓
Health security capacity				✓	✓
Citizen and community engagement and acceptance of public health guidance			✓	✓	
Public health funding					✓
Social development			✓		
Trust in the government		✓			

*Long version of the instrument, *n* = 16 interviewees. NCDs = noncommunicable diseases. The similarities between factors described overall and by country are shown by color-coding factors with a similar hue.

### 3.2 Facilitators, barriers, and lessons learned from pandemic PPR

**Figure 2:**
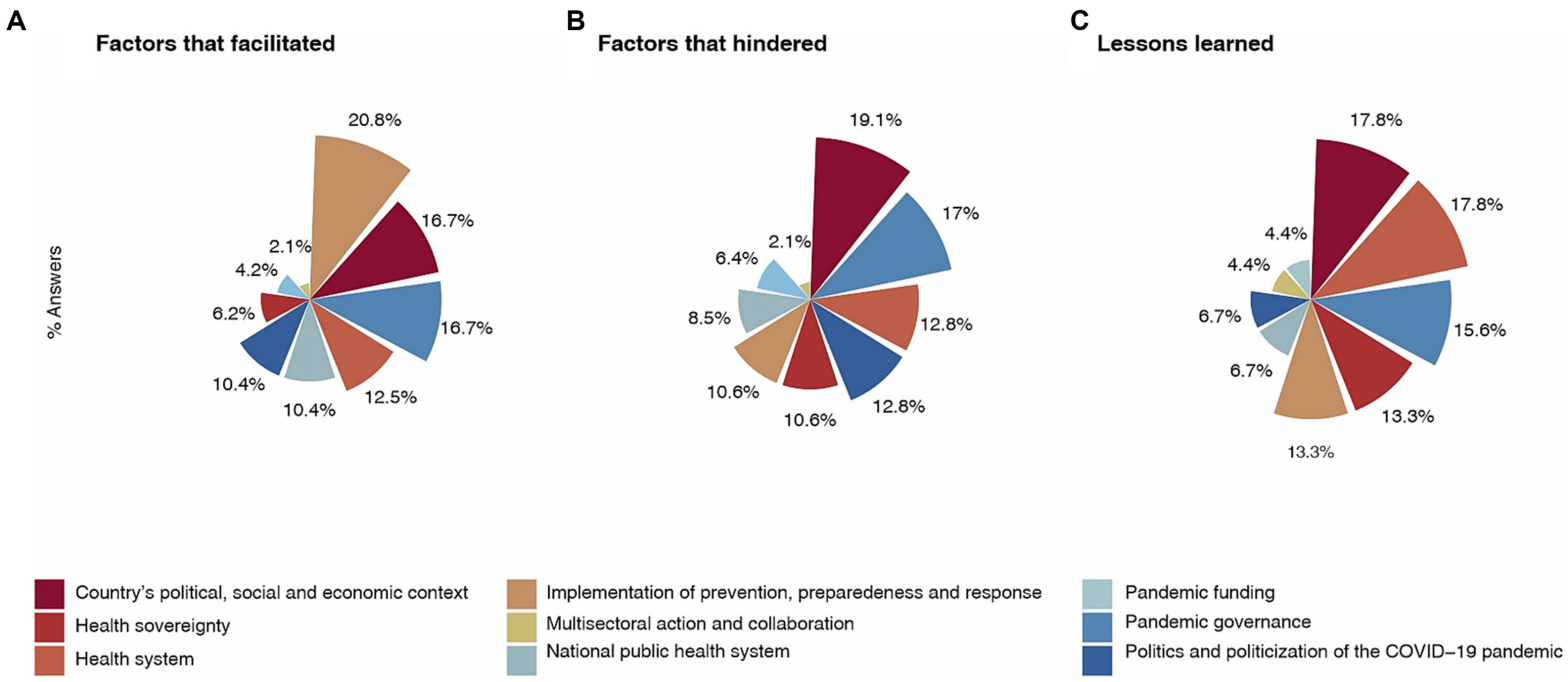
Factors that **(A)** Facilitated, **(B)** Hindered, and **(C)** led to Lessons Learned for Pandemic PPR: 2020–2021.

Having a written national pandemic plan was perceived as the most relevant facilitators (20.8%) for pandemic PPR actions, while not understanding or addressing the country’s political, social, and economic environment (19.1%) was the main perceived barrier to pandemic PPR (see Figure 2 and Supplementary material S7).

### 3.3 Experts’ perceptions of pandemic PPR by thematic category

Verbatim participant responses to the survey instrument were used to illustrate each theme.

#### 3.3.1 Theme 1: health sovereignty

To combat the COVID-19 pandemic, health sovereignty must become a priority in the region.

“We have to seek sanitary sovereignty… The region in general needs to invest more in research and development, in order to be at the forefront to be able to respond to anything that comes along. By this I do not mean that all countries should have the capacity to develop vaccines, but they should have the capacity for innovation.”

“Brazil should have been the regional leader in the response to the pandemic; indeed, regarding the supply of vaccines with Fiocruz and with the Butantan Institute, we could have manufactured and distributed vaccines in the country and in South America, but we resigned from this role because of political decisions. We are in a region where few countries have the capacity to produce goods, and we could have bought them quickly, made associations and distributed the vaccines, and we could have modified all the incidence data in the neighboring countries.”

“The potential we have in the region to operate as a block due to the easiness of language and proximity is lost if we do not capitalize on diplomatic muscle. Each country bought its vaccines alone when there was the potential to negotiate as a block.”

#### 3.3.2 Theme 2: politics and politicization of the COVID-19 Pandemic

Most participants believed the COVID-19 pandemic was politicized because countries’ politicians used the pandemic to advance their electoral and legislative goals, which included prioritizing the economy over the health of the population.

“[In Brazil] the appropriation of the COVID-19 pandemic and related health issues by political parties and electoral interests was extremely harmful.”

“Fake news is a factor that brutally hinders because it not only works specifically to misinform about COVID-19, but it also undermines trust in institutions, having medium- and long-term effects as health authorities are discredited.”

#### 3.3.3 Theme 3: multisectoral action and collaboration; most frequently public, private, academic, and philanthropic alliances or partnerships

Multisectoral collaboration was critical to manage pandemic PPR, and the private sector was an important actor that complemented mitigation efforts.

“Health cannot be managed by the health sector alone. The pandemic made it absolutely clear that if there is no health, the city does not function, and for the city to function, all sectors must be communicating and collaborating.”

“The lack of communication between the political and health organizations had a strong influence on the decisions that were made to address the problems.”

“[In Colombia] I believe that the private sector made an important contribution, not only financially, but also in terms of providing access to services such as screening and vaccination.”

“In general, there is a lot of innovation in the private sector that can be used as leverage for the public sector, and I believe that broader collaboration between the sectors could contribute to developing the countries’ health agendas and the 2030 agenda.”

#### 3.3.4 Theme 4: countries’ political, social, and economic contexts

Pandemic PPR was hindered by countries’ political, social, and economic contexts. Countries failed to address economic inequality, poverty, and job informality, and the public exhibited low levels of public trust in leaders and institutions.

“The pandemic has not been a neutral social event; it demonstrated structural inequities for very poor vulnerable groups (undocumented immigrants, Venezuelans in Latin America, and rural indigenous people). We must have universal, inclusive, resilient, and comprehensive health systems.”

“The economic outcome of the pandemic has a great impact on how the pandemic evolves, and this can be seen clearly in the region. That is, where there is a lot of job informality and a lot of instability, the poor households had to go out to work in order to live, to survive. A country with more fiscal capacity could have subsidized them while they stayed in their homes.”

“For COVID-19 deaths in particular, it is a combination of the epidemiological transition in the region, from acute conditions to chronic diseases and noncommunicable diseases in general. Latin America has dense populations, is exceedingly urban, and multigenerational, and that is a fatal combination for COVID-19.”

“[In Peru] during the pandemic, from the beginning until now [November 2022], the country has had three congresses, five presidents, and we are already on our 16th minister of health. This polarization is expressed in the distrust that citizens have in the government, so it was very difficult and continues to be difficult to unify the country behind a single purpose.”

#### 3.3.5 Theme 5: pandemic governance

Pandemic governance was hindered by mis- and disinformation and uncertainty surrounding the epidemiological development of COVID-19.

“The supranational architecture, infrastructure of agreements, treaties, and agencies was weak—the World Bank, the Inter-American Development Bank, the Pan-American Health Organization, the World Health Organization. They were ineffective to help countries with reliable information on what to do, in negotiations for purchases, in the exchange of experiences and mobilization of intangible knowledge.”

“Making sure that appropriate information got to the populations in good time and preventing or countering misinformation was very difficult for many countries. This is partially due to new forms of communication such as social media, where misinformation spread rapidly. It was partially due to governments’ perhaps not having good information or sharing incomplete information, especially early in the pandemic, which led people into behaviors that were at best ineffective and at worst counterproductive.”

“Evidence should drive policy, not politics, and instead in most countries in Latin America, there are examples of political considerations driving health policy rather than evidence from public health scholarship or practice driving public policy. Putting systems in place to try to ensure more evidence-based policy would be tremendously helpful, and most countries don’t seem to have that.”

“There is a need to formalize the interface between science and policy as well as enhance knowledge transfer and research.”

#### 3.3.6 Theme 6: implementation of PPR plans

Having political will that supports evidence-based guidance facilitated pandemic PPR. In contrast, uncoordinated responses, discounting of the future, and a lack of trust in government hindered it.

“National leadership or stewardship was absent and counterproductive for hundreds of millions of people. The role of leadership is relevant here because even a very capable health system like Brazil’s or Mexico’s, at least for middle-income countries, was unprepared for a leader who was going to countermand normal public health practice.”

“Causing economic and mental suffering [e.g., lockdowns] without a plan in place to alleviate the public health crisis is not the appropriate way to combat a pandemic. Rapid prioritization of health care attention accompanied by political leadership are priorities.”

“The region is underprepared for disasters because in general it is not giving much weight to the future when making public policy. The effectiveness of any public policy measure depends on the people and their trust, and this is where the region will face additional challenges.”

#### 3.3.7 Theme 7: pandemic funding

Increasing state funding for PPR activities and raising awareness of the post COVID-19 health consequences were lessons learned over the course of the pandemic.

“Having fiscal space to finance any measures to mitigate the impact of the crisis would have made the pandemic more bearable in the region.”

“Many countries were severely harmed politically during the pandemic, and they are attempting to reclaim lost political capital, based on economic and traditional policies (trying to improve employment, salaries), but there is a context of high inflation and problems in the global supply chain. I think they are not very interested about when the next pandemic is going to happen.”

#### 3.3.8 Theme 8: disease surveillance systems

The national public health system was strengthened by disease surveillance systems and their capacity for decision making in some countries but weakened by its absence in others.

“[In Argentina] mass testing and contact tracing could allow for early victories that did not require mass lockdown. In many cases, the lockdowns were interminable—they went on forever. Therefore, the lockdowns then become almost inconsequential, they just delay the cases, and they serve no purpose other than to cause collateral damage and did not guarantee appropriate testing and tracing measures in place.”

“Access updated data in terms of health and economic indicators in the region is very important. Due to the lack of these data, it was difficult to analyze the population in terms of social differences and determine who needed help.”

#### 3.3.9 Theme 9: fragmentation in health systems

A fragmented health system structure made pandemic management difficult, while having resilient health systems facilitated management.

“In much of the region, the health systems were weak and overwhelmed. Even in health systems that offer some form of universal access, universal coverage, the capacity was still fairly low. This was something to be expected in low-income countries, but the hope was that middle-income countries would be more resilient.”

“The precariousness, fragmentation, and decentralization of the health system in Peru left the Minister of Health with very little power.”

Respondents did not see an optimistic future for epidemic and pandemic PPR. Respondents’ perceptions of national decision-makers were divided between seeing the decision-makers as only somewhat supportive or not at all supportive of improving each country’s pandemic PPR in the next 12 months (see Supplementary material S8). Perceptions varied by country (see Supplementary material S9). In Argentina, participants perceived national decision makers as somewhat supportive. National decision makers in Colombia were perceived as not very supportive of pandemic PPR, and less than a fifth of participants perceived them as very supportive. In Brazil and Peru, more than three-fourths of decision makers were perceived as not at all supportive.

The most trustworthy bodies and sectors for national pandemic PPR decision-making in the next 1–2 years varied by country (see Supplementary material S10). In general, respondents deemed academia, health associations, national ministries and institutes, regional authorities, organizations, and organisms important for future decision-making. Participants’ rankings of health authorities and policymakers’ top priorities for the coming year were influenced by contextual characteristics in each country (see Supplementary material S11). Nonetheless, several themes were shared across countries: the expansion and reform of health care systems, investment in health infrastructure (e.g., laboratories, technologies, human resources, etc.), pandemic preparedness, the inclusion of vulnerable groups in the health agenda, and epidemiological surveillance and research.

## 4 Discussion

The COVID-19 pandemic occurred in a setting of inadequate preparedness, lack of coordinated responses, and vacuum of global, regional, national, and community leadership. South American countries such as Brazil and Peru were among the world’s hardest hit by the pandemic. Hence, this study is unique as it not only draws on the opinions of high-level experts and decision makers to examine the PPR response to the COVID-19 pandemic in Argentina, Brazil, Colombia, and Peru, but also makes recommendations to increase PPR to manage similar emerging health threats based on the specific region’s political, social, and economic context.

Our mixed methods approach revealed that (a) considerable variation in policy response across countries, particularly in the early response to the pandemic due to political context and will. These are essential for sustaining evidence-based guidance and health sovereignty, especially during a regional public health emergency of unprecedented magnitude and impact; (b) the region’s volatile political environment requires the construction of robust and independent public health institutions to prevent political ideology from influencing the policy response to national health emergencies. Countries should capitalize on past successes managing infectious disease outbreaks and the region’s strong immunization tradition; (c) the absence of regional leadership, multilateralism, and coordinated multisectoral action squandered the region’s capacity to negotiate as a bloc for public goods procurement, vaccine purchases, and manufacturing, distribution, and investment in science, technology, and infrastructure to develop capacities to manage a new crisis; (d) the region’s disproportionately high disease burden resulted from the failure to understand the local context, which includes unequal access to healthcare, weaknesses in PHC, under-resourced health care systems, high poverty and labor informality, socioeconomic inequities, poor screening, isolation, and tracking of patients; (e) ineffective risk communication and community engagement strategies, scientific denialism, medical misinformation, and a lack of pandemic knowledge compromised public trust and hindered pandemic governance; and (f) post-COVID-19 recovery programs require trustworthy and competent government and private institutions.

These findings are consistent with recent research from Brazil and Mexico indicating how politics disabled a science- and evidence-based response and reduced the public health structure and health system functionality to undertake pandemic PPR efforts (20). Also, evidence of Latin American subnational policy responses throughout the first year of the pandemic show how important a coordinated national policy response is for engaging subnational governments and adjusting local responses to individual settings with a health equity perspective (7). Given the region’s high population density, high informal employment, poverty, and a high burden of NCDs, evidence demonstrates how useful it is to apply a multisectoral, inter-institutional approach that takes advantage of and engages formal and informal networks and all available actors in PPR activities (21). For example, the successful case of the program “Companies for Vaccination” (Empresas por la Vacunación) which was the first corporate mass vaccination program in the world led by the National Association of Entrepreneurs of Colombia ANDI (22).

In addition, experts highlight the importance of national health security capabilities, based on a long history of developing epidemiological surveillance systems for outbreak control and the mitigation of morbidity and mortality during pandemics (23). Management of infectious diseases like Zika, dengue, chikungunya, malaria, and influenza have also promoted the evolution of coordinated intersectoral planning and response systems in the region (23). Aside from the political context, these findings show that countries’ ability to respond to public health emergencies depends on their coordination of scientific evidence, ability to follow international health regulations, and deployment of a social determinants of health approach that implements strategies based on regional characteristics (e.g., poverty, job informality, and food insecurity). The latter limit the effective implementation of outbreak control and mitigation interventions such as quarantines, contact tracing, and social isolation (24, 25).

Despite these findings, decision-making based on long-term planning is unusual in South America, both in general and for PPR (26, 27). As a result of post pandemic competing interests and electoral strategies in 2022, policymakers lost focus on evidence-based public health action and did not prioritize pandemic PPR in their countries’ public health agendas for 2023. Three years after the onset of the COVID-19 pandemic, this study shows that South American policymakers still do not prioritize pandemic PPR in their health agendas. Systemic corruption, politically driven messages, multidimensional inequality, and social media misinformation obstructed ongoing pandemic PPR efforts and will continue to do so if no measures are taken.

Beyond South America, other LMICs in Africa and South-East Asia faced similar pandemic PPR challenges revealing the global nature of the problem (28–30). The Lancet COVID-19 Commission (31) and the Series on One Health and Global Health Security (30) recommended that in the fight against infectious diseases, countries must carry out prevention, containment, global innovation, and diffusion across health systems that include equitable protection of the population, measures to protect vulnerable groups, and improvements in One Health institutional and professional capacities. In addition, the Independent Panel for Pandemic Preparedness and Response (32) highlighted that political will and commitment were key elements of public policy effectiveness. Richmond and Kotelchuck’s conceptual framework (33) lists political will as one of the three key factors of robust public policy, along with a scientific knowledge base and social strategies. Similarly, greater political will and creative communication are important factors that can promote and expand global health funding (34). Thus, prominent governmental and financial entities, well-known activists, local leaders, and the global and regional media must be included, empowered, and informed about PPR and its importance for public health and the post-COVID-19 pandemic period to strengthen pandemic PPR efforts in South America.

International consensus exists on the need to elevate PPR in global public policy and public health agendas. This study’s expert group recommends the following specific PPR actions for mitigating the health burden of the COVID-19 pandemic in South America and other LMICs, and for enacting future pandemic PPR:

1. The creation of a regional task force on COVID-19, post-pandemic recovery, and emerging disease threats to hold governments accountable for the PPR agenda in five years. This group should be comprised of actors from governments, nongovernmental organizations, academia, health, private, philanthropic, and multilateral sectors, and PAHO. The task force should also oversee post-pandemic plans and protocols to boost PHC and health care system capacities (physical and mental rehabilitation for COVID-19 survivors), and ensure equal access to vaccines and medical devices (35).
2. Improvement of surveillance systems: a) investing in metagenomics and molecular diagnostics to identify emergent disease events and ensure an etiological, not syndromic, diagnosis that can prompt health security warnings and epidemic investigations, even in remote regions. A large, well-trained health personnel with adequate working conditions is needed to develop these capabilities; b) investing in information systems that produce and distribute open, transparent, real-time, and reliable health data can help decision makers affect risk-informed changes in population behavior; c) investing in mathematical modeling can help policymakers make decisions surrounding outbreak alerts and health security when data is ambiguous.
3. The development of social media platforms to prioritize and invest in risk communication strategies, community engagement to combat mis- and dis-information, vaccination hesitancy, and scientific denialism, especially in extremely underserved populations.
4. Improvements in social and economic protection for vulnerable population groups (e.g., Indigenous Peoples, afro-descendants, remote rural communities, low-income households, migrants).

These recommendations reflect our conclusions about the emerging priority areas for action in infectious disease emergency preparedness and are supported by the findings of a recent scoping review (36). These include the need for health systems planning, improvements in epidemiologic surveillance and monitoring, laboratory systems for infectious disease preparedness and response, collaborative research and health policy networks in the global south, risk communication and health pedagogy, governance and leadership, increased resources, local capacity, and community engagement.

This study has strengths and limitations that deserve attention. Key respondents represented 19 organizations in South America’s four most populous countries and five multilateral organizations in South America showing the regional relevance of the study (thematic saturation was achieved with the study’s sample). Limitations include that not all South American countries were included in this study, much less all countries within the Latin American and Caribbean region. In addition, Brazilian government officials did not contribute to the data in our analysis. As such, data may not reflect the entire spectrum of key informants and stakeholders involved in pandemic management.

## 5 Conclusion

The COVID-19 pandemic laid bare policymaking deficiencies at all levels of government, but especially the vulnerability of health systems to politicization, mis- and dis-information in Latin America as well as other LMICs. Additionally, a lack of health infrastructure, support for vulnerable populations, and capacity to deliver services harmed the pandemic response in all four countries.

This study demonstrates that three years after the COVID-19 pandemic began, policymakers at the highest levels of government in Argentina, Brazil, Colombia, and Peru are not prioritizing pandemic PPR in their countries’ health agendas. To place pandemic PPR at the top of Argentina, Brazil, Colombia, Peru, and South American national public health agendas, we recommend establishing a regional task force on COVID-19, post-pandemic recovery, and emerging disease threats in the next two years, improving social and economic protection for vulnerable groups, improving primary health care, disease surveillance, and information systems, and developing systems for risk communication and community engagement to improve pandemic PPR.

## Data availability statement

The raw data supporting the conclusions of this article will be made available by the authors, without undue reservation.

## Ethics statement

The studies involving humans were approved by Ethics approval was obtained from the Ethics Committee of Universidad de los Andes (2022; Approval No. 20220401). The studies were conducted in accordance with the local legislation and institutional requirements. The participants provided their written informed consent to participate in this study.

## Author contributions

ARV: Conceptualization, Formal analysis, Funding acquisition, Investigation, Methodology, Project administration, Writing – original draft, Writing – review & editing. MT: Writing – original draft, Writing – review & editing. JJM: Writing – review & editing. JMG: Formal analysis, Investigation, Methodology, Writing – original draft, Writing – review & editing. RL: Writing – review & editing. GC: Writing – review & editing. MF: Writing – review & editing. AG: Funding acquisition, Resources, Writing – review & editing. AMOH: Funding acquisition, Resources, Writing – review & editing. EOVD: Investigation, Methodology, Writing – review & editing. AVM: Investigation, Methodology, Writing – review & editing. NMVR: Writing – review & editing. SRR: Funding acquisition, Resources, Writing – review & editing.
