# Supplementary material for: Assessing pandemic preparedness, response, and lessons learned from the COVID-19 pandemic in four south American countries: agenda for the future
Source: Front Public Health. 2023 Nov 29;11:1274737. doi: 10.3389/fpubh.2023.1274737 (PMC10716458; doi:10.3389/fpubh.2023.1274737)
Supplement: Supplementary file 1 [file Data_Sheet_1.docx]

Supplementary Material

# S1. Key Informant Interview (Long version) - Assessing pandemic preparedness, response and lessons learned from the COVID-19 pandemic in Latin America.

***Opening***

Thank you for agreeing to participate in this project and answer to this interview. You were selected because you are a government contact at the national/subnational level, a member of academia or a multilateral organization and you were involved in the country's response during the first two years of the COVID-19 pandemic.

Your participation in this survey is voluntary. While it is important that you can answer all questions, it is important to clarify that you are not required to do so and may withdraw at any time.

The survey will take approximately 60 minutes to complete, depending on your answers.

As stated in the email sent, we would like your permission to record this interview. Your name will not be linked to anything said during our conversation today. There are no right or wrong answers, and we appreciate all your feedback.

***Data gathering***

*In an effort to better understand the management of the COVID-19 pandemic in Latin America, lessons learned and recommendations for the future, I would like to learn more about your experiences and opinions during the first two years of the COVID-19 pandemic in: Argentina, Brazil, Chile, Colombia or Peru.*

*The interview will gather data on five blocks:*

1. ***General information:*** *we will collect your names, surnames, and institutional affiliation.*
2. ***National pandemic preparedness plan:*** *we will assess the COVID-19 pandemic management based on the presence of a pandemic preparedness and response plan.*
3. ***Implementation of pandemic preparedness and response indicators, the social, political and economic context of the country, and health outcomes:*** *we will assess perceptions of the correlation between implementation of the COVID-19 pandemic preparedness and response plan, political, social, economic and health factors, and health outcomes (total number of cases, deaths and vaccination coverage).*
4. ***Lessons learned from COVID-19 pandemic preparedness and response during 2020-2021:*** *we will assess the perception of how the country's political, economic, social, trust, health system, internal and external factors facilitated and hindered the implementation of the COVID-19 pandemic preparedness and response plan.*
5. ***Recommendations for the future of epidemic and pandemic preparedness and response in the country:*** *we will gather information on the country's future agenda for preparedness and response to future epidemics and pandemics, as well as the involvement and support of governmental and non-governmental organizations.*

***Block 1: General information***

| **Name:** |
| --- |
| **Surname:** |
| **Country for which you are providing responses:** |
| - Argentina - Brazil - Chile - Colombia - Peru |
| **Where do you work?** |
| **Name of the institution:** |
| **Select the best description for your institution:**   - Government office at national/subnational level involved in the response to the COVID-19 pandemic during 2020-2021 - Academic body involved in the response to the COVID-19 pandemic during 2020-2021 - Multilateral organization involved in the response to the COVID-19 pandemic during 2020-2021 - **Philanthropic sector** |
| **What is your official role (position/title)?** |
|  |
| **How long have you been in this position?** |
|  |

***Block 2: National pandemic preparedness plan***

*First, we would like to hear about your own experience and views on the COVID-19 pandemic preparedness and response in [COUNTRY].* ***For the purpose of this interview, and according to the United Nations and World Health Organization “preparedness is the ability of governments, professional response organizations, communities, and individuals to anticipate, detect, and respond effectively to, and recover from, the impact of likely, imminent, or current health emergencies, hazards, events, or conditions. It means putting in place mechanisms that will allow national authorities, multilateral organizations, and relief organizations to be aware of risks and deploy staff and resources quickly once a crisis strikes”.*** *Please, think about your own experiences, and views when answering the following questions.*

**1. How would you describe your involvement in the preparedness and response to the COVID-19 pandemic in your country? Could you briefly talk about this experience?**

- - *Probe:* Did you ever work in any health-related agency or government institution involved in the response to the COVID-19 pandemic, or in any other policy or practice setting?

|  |
| --- |

*For question 2 we require the reference of the document in case it exists and if you could share it with us if available. We will not ask questions related to the content of the document.*

**2. Do you know if your country has a COVID-19 pandemic preparedness and response plan?**

- Yes
- No

If the answer is yes, please indicate:

| Plan/Document Title: |  |
| --- | --- |
| Web link: |  |

***Block 3: Implementation of pandemic preparedness and response indicators, the social, political and economic context of the country, and health outcomes***

*For the following questions, you will have a document available with the most updated estimates of the health outcomes of interest in this study (number of cases, deaths and vaccination coverage) in [COUNTRY].*

*It is estimated that there are about 504 million cases of COVID-19 and more than 6.2 million deaths worldwide, with the Latin American region accounting for 14% of the total number of cases and 28% of the total number of deaths in the world. A disproportionate representation considering that these countries account for less than 9% of the world's population.*

**3. In your opinion, to what extent do each of the following pandemic preparedness and response items (national plan implementation, if available) relate to current pandemic outcomes in [COUNTRY] in 2020-2021?** *(Adapted table from Heymann, David L., et al. "Global health security: the wider lessons from the west African Ebola virus disease epidemic." The Lancet 385.9980 (2015): 1884-1901.)*

| **Item** | **Outcomes** | **Highly correlated** | **Somewhat correlated** | **Somewhat uncorrelated** | **Highly uncorrelated** |
| --- | --- | --- | --- | --- | --- |
| **Prevention activities** | | | | | |
| Attention to zoonotic diseases reported in surveillance systems. | Incidence |  |  |  |  |
|  | Deaths |  |  |  |  |
|  | Vaccination rates against COVID-19 |  |  |  |  |
| Bioprotection and biosecurity measures, systems, training activities to ensure protection and safety to handle biological material. | Incidence |  |  |  |  |
|  | Deaths |  |  |  |  |
|  | Vaccination rates against COVID-19 |  |  |  |  |
| Regular immunization programs. | Incidence |  |  |  |  |
|  | Deaths |  |  |  |  |
|  | Vaccination rates against COVID-19 |  |  |  |  |
| **Detection and reporting activities** | | | | | |
| Existence of a national laboratory system with capacity and quality for the detection of priority diseases, referral and transport of samples. | Incidence |  |  |  |  |
|  | Deaths |  |  |  |  |
|  | Vaccination rates against COVID-19 |  |  |  |  |
| Detection and notification of events of public health interest with surveillance systems for real-time detection. | Incidence |  |  |  |  |
|  | Deaths |  |  |  |  |
|  | Vaccination rates against COVID-19 |  |  |  |  |
| Accessibility and transparency of epidemiological surveillance data. | Incidence |  |  |  |  |
|  | Deaths |  |  |  |  |
|  | Vaccination rates against COVID-19 |  |  |  |  |
| Real time surveillance systems’ report, data accessibility and transparency. | Incidence |  |  |  |  |
|  | Deaths |  |  |  |  |
|  | Vaccination rates against COVID-19 |  |  |  |  |
| Development and training of personnel for the health system (basic capacities). | Incidence |  |  |  |  |
|  | Deaths |  |  |  |  |
|  | Vaccination rates against COVID-19 |  |  |  |  |
| **Response activities** | | | | | |
| Emergency operations centers and/or early warning systems for the detection of public health events. | Incidence |  |  |  |  |
|  | Deaths |  |  |  |  |
|  | Vaccination rates against COVID-19 |  |  |  |  |
| Multisectoral response and risk communication. | Incidence |  |  |  |  |
|  | Deaths |  |  |  |  |
|  | Vaccination rates against COVID-19 |  |  |  |  |
| Medical countermeasure and health personnel deployment. | Incidence |  |  |  |  |
|  | Deaths |  |  |  |  |
|  | Vaccination rates against COVID-19 |  |  |  |  |
| Installed capacity, supply chain and medical care in clinics, hospitals, care centers. | Incidence |  |  |  |  |
|  | Deaths |  |  |  |  |
|  | Vaccination rates against COVID-19 |  |  |  |  |

**4. In your opinion, to what extent did each of the following factors facilitate or hinder pandemic preparedness and response/plan implementation during the first two years of the COVID-19 pandemic in [COUNTRY]?**

*For the following questions, you will have available a specific profile for your country with the most updated data for each of the indicators.*

| **Political, sociodemographic and economic** | | | | | | |
| --- | --- | --- | --- | --- | --- | --- |
|  | **Factor that influenced** | | **Facilitated** | **Somewhat facilitated** | **Somewhat hindered** | **Hindered** |
| 1 | Sociodemographic context | Population (2021) |  |  |  |  |
|  |  | Life expectancy in years (2019) |  |  |  |  |
|  |  | Human Development Index (2019) |  |  |  |  |
|  |  | Adult literacy rate (2020) |  |  |  |  |
| 2 | Political unrest | State Fragility Index (2020) |  |  |  |  |
|  |  | Corruption Perception Index (2020) |  |  |  |  |
| 3 | Poverty and informal employment | GINI Index (2019) |  |  |  |  |
|  |  | Poverty (2020) |  |  |  |  |
|  |  | Extreme poverty (2020) |  |  |  |  |
|  |  | Proportion of informal employment in total employment (most recent year) |  |  |  |  |
| **Public health context** | | | | | | |
| 4 | National health security capacity | GHS Index overall score (2021) |  |  |  |  |
| 5 | National health system structure/ Universal Health Care | UHC service coverage index (most recent year) |  |  |  |  |
| 6 | National health system capacity/resources | Hospital beds per 1000 population (most recent year) |  |  |  |  |
|  |  | Doctors per 1000 population (most recent year) |  |  |  |  |
|  |  | Nurses per 1000 population (most recent year) |  |  |  |  |
| 7 | Public health funding during the pandemic | % of GDP (approx.) (2021) |  |  |  |  |
| 8 | COVID19 Misinformation | Percentage of inability to recognize fake news of COVID-19 (2020) |  |  |  |  |
| 9 | Technical, technological and industrial capacity of the country to manufacture vaccines | Capacity to produce vaccines in development |  |  |  |  |
| 10 | Vaccines access and availability during the pandemic | Based on experience |  |  |  |  |
| **Population’s health** | | | | | | |
| 11 | % Deaths due to NCDs and risk factors prevalence | % Deaths by non-communicable diseases (2019) |  |  |  |  |
|  |  | Prevalence of overweight and obesity in adults (%) (2016) |  |  |  |  |
| **Relationship between citizens and institutions** | | | | | | |
| 12 | Public trust/confidence in the government | Public trust/confidence in the government (2020) |  |  |  |  |
| 13 | Public trust/confidence in health institutions | On a scale of 1 (no trust at all) to 7 (a lot of trust): Public trust in the health care system (2022) |  |  |  |  |
| 14 | Public trust/confidence in public leaders | On a scale of 1 (no trust at all) to 7 (a lot of trust): Public trust in the mayor, public trust in the governor, public trust in the president (2022) |  |  |  |  |
| 15 | Public trust/confidence in scientists and scientific evidence | Public trust/confidence in scientists and scientific evidence (2020) |  |  |  |  |
| 16 | Citizen and community engagement | Generalized trust (2022) |  |  |  |  |

**5. Based on the two previous questions, in your opinion, which were the three most important factors for pandemic preparedness and response in your country during 2020-2021 in [COUNTRY] or [REGION]? Why? (Open-ended question)**

| 1. |  |
| --- | --- |
| 2. |  |
| 3. |  |

**6. Based on the two previous questions, in your opinion, which were the three factors that were most difficult to manage in the pandemic preparedness and response in your country during 2020-2021 in [COUNTRY] or [REGION]? Why? (Open-ended question)**

| 1. |  |
| --- | --- |
| 2. |  |
| 3. |  |

***Block 4: Lessons learned from COVID-19 pandemic preparedness and response***

**7. In** **your opinion, which are the top three lessons learned about pandemic preparedness and response in [COUNTRY] or [REGION] during 2020-2021? (Open-ended question)**

| 1. |  |
| --- | --- |
| 2. |  |
| 3. |  |

***Block 5: Future pandemic preparedness and response***

**8. Think broadly about the current national public health policy setting in [COUNTRY] or [REGION] Which do you think are the top three areas/priorities that health authorities and policymakers will address in the next 12 months?**

| 1. |  |
| --- | --- |
| 2. |  |
| 3. |  |

**9. To what extent would you say national decision-makers in [COUNTRY] are supportive of focusing on [epidemic and pandemic preparedness and response] in the next 12 months? Would you say they are:**

| **Select one** | | **Why?** |
| --- | --- | --- |
| Very supportive |  |  |
| Somewhat supportive |  |  |
| Not very supportive |  |  |
| Not at all supportive |  |  |

**10. Which are the three most prominent organizations in [COUNTRY] do you see as key/credible for [epidemics and pandemics preparedness and response] decision making at the national level for the next 1-2 years?**

| 1. |  |
| --- | --- |
| 2. |  |
| 3. |  |

**11. Finally, is there anything else you would like to add about how the political, socioeconomic and health system contexts influenced the preparedness and response for the management of the COVID-19 pandemic during 2020-2021 and, consequently, may have changed health outcomes (incidence, deaths and vaccination coverage) in [COUNTRY] or [REGION]?**

|  |
| --- |

***Thank you for your time and input. Those are all the questions I have.***

# S2. Key Informant Interview (Short version) - Assessing pandemic preparedness, response and lessons learned from the COVID-19 pandemic in Latin America.

**Dr.**

Thank you for agreeing to participate in this project and to answer this interview. You have been invited to participate as a government contact at the national or international level, member of the academia or of a multilateral organization, that participated in the COVID-19 pandemic response. Your participation in this survey is voluntary. You are not required to answer all questions, and you may withdraw from the survey at any time. However, it would be vital if you could answer all the questions in the survey. The survey will take approximately 15-20 minutes to complete, depending on your responses.

Your name will not be linked to anything said during our conversation today. There are no right or wrong answers, and we appreciate all of your feedback.

In an attempt to better understand the factors that influenced the COVID-19 pandemic management in the region, lessons learned, and recommendations, I would like to learn more about your views and experience during the first two years of the COVID-19 pandemic in the Latin American region. For this interview, we will request you to please provide insights and examples from other Latin American countries’ pandemic preparedness and response during 2020-2021.

*For the purpose of this interview, and according to the United Nations and World Health Organization “preparedness is the ability of governments, professional response organizations, communities, and individuals to anticipate, detect, and respond effectively to, and recover from, the impact of likely, imminent, or current health emergencies, hazards, events, or conditions. It means putting in place mechanisms that will allow national authorities, multilateral organizations, and relief organizations to be aware of risks and deploy staff and resources quickly once a crisis strikes”. Please, think about your own experiences, and views when answering the following questions.*

***Please complete the following data:***

| **Name:** |
| --- |
| **Where do you work?** |
| **What is your official role (position/title)?** |
|  |
| **How long have you been in this position?** |
|  |

***Questionnaire***

**1. To your knowledge, could you please list the three most important factors for the COVID-19 pandemic preparedness and response in Latin America during 2020-2021 and why? Are there any examples you would like to share with us?**

| 1. |  |
| --- | --- |
| 2. |  |
| 3. |  |

**2. To your knowledge, could you please list the three factors that were the most difficult to manage for the COVID-19 pandemic preparedness and response in Latin America during 2020-2021 and why? Are there any examples you would like to share with us?**

| 1. |  |
| --- | --- |
| 2. |  |
| 3. |  |

**3. In your opinion, which were the three main lessons learned about pandemic preparedness and response in Latin America during 2020-2021 and why? Are there any examples you would like to share with us?**

| 1. |  |
| --- | --- |
| 2. |  |
| 3. |  |

**4. In your opinion, what do you think will be the top three public health policy areas that health authorities will prioritize in Latin America in the next 12 months? Why?**

| 1. |  |
| --- | --- |
| 2. |  |
| 3. |  |

**5. Finally, is there anything you would like to add to help me understand your thoughts about how the COVID-19 pandemic preparedness and response during 2020-2021 in Latin American countries were affected by political, socio-economic, and health system contexts and may have shaped pandemic trajectories and outcomes (incidence, deaths, and vaccination coverage)?**

|  |
| --- |

***Thank you for your time and input. Those are all the questions I have.***

# S3. Perception of Pandemic Prevention, Preparedness and Response Items Related to COVID-19 Incidence.*

| **Item** | **Highly correlated** | | **Somewhat correlated** | | **Somewhat uncorrelated** | | **Highly uncorrelated** | |
| --- | --- | --- | --- | --- | --- | --- | --- | --- |
|  | n | % | n | % | n | % | n | % |
| Accessibility and transparency of epidemiological surveillance data | 13 | 81.3% | 2 | 12.5% | 1 | 6.3% | 0 | 0.0% |
| Attention to zoonotic diseases reported in surveillance systems | 7 | 43.8% | 4 | 25.0% | 3 | 18.8% | 2 | 12.5% |
| Bioprotection and biosecurity measures, systems, training activities to ensure protection and safety to handle biological material | 10 | 62.5% | 4 | 25.0% | 0 | 0.0% | 2 | 12.5% |
| Detection and notification of events of public health interest with surveillance systems for real-time detection | 14 | 87.5% | 2 | 12.5% | 0 | 0.0% | 0 | 0.0% |
| Emergency operations centers and/or early warning systems for the detection of public health events | 10 | 62.5% | 4 | 25.0% | 0 | 0.0% | 2 | 12.5% |
| Existence of a national laboratory system with capacity and quality for the detection of priority diseases, referral and transport of samples | 13 | 81.3% | 2 | 12.5% | 1 | 6.3% | 0 | 0.0% |
| Installed capacity, supply chain and medical care in clinics, hospitals, care centers | 6 | 37.5% | 7 | 43.8% | 2 | 12.5% | 1 | 6.3% |
| Medical countermeasure and health personnel deployment | 9 | 56.3% | 4 | 25.0% | 3 | 18.8% | 0 | 0.0% |
| Multisectoral response and risk communication | 11 | 68.8% | 4 | 25.0% | 1 | 6.3% | 0 | 0.0% |
| Real time surveillance systems’ report, data accessibility and transparency | 10 | 62.5% | 5 | 31.3% | 1 | 6.3% | 0 | 0.0% |
| Regular immunization programs | 5 | 31.3% | 6 | 37.5% | 2 | 12.5% | 3 | 18.8% |

* Long version of the instrument, n = 16 interviewees.

# S4. Perception of Pandemic Prevention, Preparedness and Response Items Related to COVID-19 Deaths.*

| **Item** | **Highly correlated** | | **Somewhat correlated** | | **Somewhat uncorrelated** | | **Highly uncorrelated** | |
| --- | --- | --- | --- | --- | --- | --- | --- | --- |
|  | n | % | n | % | n | % | n | % |
| Accessibility and transparency of epidemiological surveillance data | 11 | 68.8% | 4 | 25.0% | 1 | 6.3% | 0 | 0.0% |
| Attention to zoonotic diseases reported in surveillance systems | 4 | 25.0% | 5 | 31.3% | 4 | 25.0% | 3 | 18.8% |
| Bioprotection and biosecurity measures, systems, training activities to ensure protection and safety to handle biological material | 7 | 43.8% | 5 | 31.3% | 2 | 12.5% | 2 | 12.5% |
| Detection and notification of events of public health interest with surveillance systems for real-time detection | 9 | 56.3% | 7 | 43.8% | 0 | 0.0% | 0 | 0.0% |
| Emergency operations centers and/or early warning systems for the detection of public health events | 6 | 37.5% | 6 | 37.5% | 3 | 18.8% | 1 | 6.3% |
| Existence of a national laboratory system with capacity and quality for the detection of priority diseases, referral and transport of samples | 8 | 50.0% | 4 | 25.0% | 3 | 18.8% | 1 | 6.3% |
| Installed capacity, supply chain and medical care in clinics, hospitals, care centers | 11 | 68.8% | 4 | 25.0% | 1 | 6.3% | 0 | 0.0% |
| Medical countermeasure and health personnel deployment | 9 | 56.3% | 5 | 31.3% | 2 | 12.5% | 0 | 0.0% |
| Multisectoral response and risk communication | 11 | 68.8% | 4 | 25.0% | 1 | 6.3% | 0 | 0.0% |
| Real time surveillance systems’ report, data accessibility and transparency | 10 | 62.5% | 4 | 25.0% | 2 | 12.5% | 0 | 0.0% |
| Regular immunization programs | 5 | 31.3% | 6 | 37.5% | 1 | 6.3% | 4 | 25.0% |

* Long version of the instrument, n = 16 interviewees.

# S5. Perception of Pandemic Prevention, Preparedness and Response Items Related to COVID-19 Vaccination.*

| **Item** | **Highly correlated** | | **Somewhat correlated** | | **Somewhat uncorrelated** | | **Highly uncorrelated** | |
| --- | --- | --- | --- | --- | --- | --- | --- | --- |
|  | n | % | n | % | n | % | n | % |
| Accessibility and transparency of epidemiological surveillance data | 11 | 68.8% | 4 | 25.0% | 1 | 6.3% | 0 | 0.0% |
| Attention to zoonotic diseases reported in surveillance systems | 4 | 25.0% | 5 | 31.3% | 4 | 25.0% | 3 | 18.8% |
| Bioprotection and biosecurity measures, systems, training activities to ensure protection and safety to handle biological material | 7 | 43.8% | 5 | 31.3% | 2 | 12.5% | 2 | 12.5% |
| Detection and notification of events of public health interest with surveillance systems for real-time detection | 9 | 56.3% | 7 | 43.8% | 0 | 0.0% | 0 | 0.0% |
| Emergency operations centers and/or early warning systems for the detection of public health events | 6 | 37.5% | 6 | 37.5% | 3 | 18.8% | 1 | 6.3% |
| Existence of a national laboratory system with capacity and quality for the detection of priority diseases, referral and transport of samples | 8 | 50.0% | 4 | 25.0% | 3 | 18.8% | 1 | 6.3% |
| Installed capacity, supply chain and medical care in clinics, hospitals, care centers | 11 | 68.8% | 4 | 25.0% | 1 | 6.3% | 0 | 0.0% |
| Medical countermeasure and health personnel deployment | 9 | 56.3% | 5 | 31.3% | 2 | 12.5% | 0 | 0.0% |
| Multisectoral response and risk communication | 11 | 68.8% | 4 | 25.0% | 1 | 6.3% | 0 | 0.0% |
| Real time surveillance systems’ report, data accessibility and transparency | 10 | 62.5% | 4 | 25.0% | 2 | 12.5% | 0 | 0.0% |
| Regular immunization programs | 5 | 31.3% | 6 | 37.5% | 1 | 6.3% | 4 | 25.0% |

* Long version of the instrument, n = 16 interviewees.

# S6. Overall factors that Facilitated or Hindered Pandemic Preparedness and Response Activities during the first two years of the COVID-19 Pandemic.

| **Factor** | **Facilitated** | | **Somewhat facilitated** | | **Somewhat hindered** | | **Hindered** | |
| --- | --- | --- | --- | --- | --- | --- | --- | --- |
| % Deaths by non-communicable diseases | 1 | 6.3% | 0 | 0.0% | 10 | 62.5% | 5 | 31.3% |
| % of GDP (approx.) | 1 | 6.3% | 6 | 37.5% | 4 | 25.0% | 5 | 31.3% |
| Adult literacy rate | 6 | 40.0% | 4 | 26.7% | 4 | 26.7% | 1 | 6.7% |
| Corruption perception index | 1 | 6.3% | 0 | 0.0% | 7 | 43.8% | 8 | 50.0% |
| Doctors per 1000 population | 3 | 18.8% | 2 | 12.5% | 5 | 31.3% | 6 | 37.5% |
| Extreme poverty | 1 | 6.3% | 1 | 6.3% | 1 | 6.3% | 13 | 81.3% |
| Generalized trust | 0 | 0.0% | 3 | 18.8% | 7 | 43.8% | 6 | 37.5% |
| GHS Index overall score | 4 | 25.0% | 4 | 25.0% | 4 | 25.0% | 4 | 25.0% |
| Gini index | 1 | 6.3% | 1 | 6.3% | 3 | 18.8% | 11 | 68.8% |
| Hospital beds per 1000 population | 4 | 25.0% | 0 | 0.0% | 7 | 43.8% | 5 | 31.3% |
| Human Development Index | 5 | 33.3% | 0 | 0.0% | 5 | 33.3% | 5 | 33.3% |
| Life expectancy in years | 2 | 13.3% | 7 | 46.7% | 5 | 33.3% | 1 | 6.7% |
| Nurses per 1000 population | 3 | 18.8% | 1 | 6.3% | 3 | 18.8% | 9 | 56.3% |
| Percentage of the population that is unable to recognize COVID-19 fake news | 0 | 0.0% | 1 | 6.3% | 7 | 43.8% | 8 | 50.0% |
| Population | 2 | 14.3% | 4 | 28.6% | 5 | 35.7% | 3 | 21.4% |
| Poverty | 2 | 12.5% | 0 | 0.0% | 4 | 25.0% | 10 | 62.5% |
| Prevalence of overweight and obesity in adults (%) | 0 | 0.0% | 1 | 6.3% | 9 | 56.3% | 6 | 37.5% |
| Proportion of informal employment in total employment | 0 | 0.0% | 1 | 6.7% | 1 | 6.7% | 13 | 86.7% |
| Public trust in science | 4 | 25.0% | 8 | 50.0% | 1 | 6.3% | 3 | 18.8% |
| Public trust in the government | 2 | 12.5% | 1 | 6.3% | 8 | 50.0% | 5 | 31.3% |
| Public trust in the health care system | 2 | 12.5% | 5 | 31.3% | 5 | 31.3% | 4 | 25.0% |
| Public trust/confidence in public leaders | 0 | 0.0% | 1 | 6.3% | 6 | 37.5% | 9 | 56.3% |
| State fragility index | 0 | 0.0% | 2 | 12.5% | 4 | 25.0% | 10 | 62.5% |
| UHC service coverage index | 5 | 33.3% | 5 | 33.3% | 3 | 20.0% | 2 | 13.3% |
| Vaccines access and availability during the pandemic Based on experience | 4 | 25.0% | 3 | 18.8% | 6 | 37.5% | 3 | 18.8% |
| Vaccines development | 3 | 18.8% | 1 | 6.3% | 5 | 31.3% | 7 | 43.8% |

* Long version of the instrument, n = 16 interviewees.

# S7. Main Themes Related to Pandemic PPR


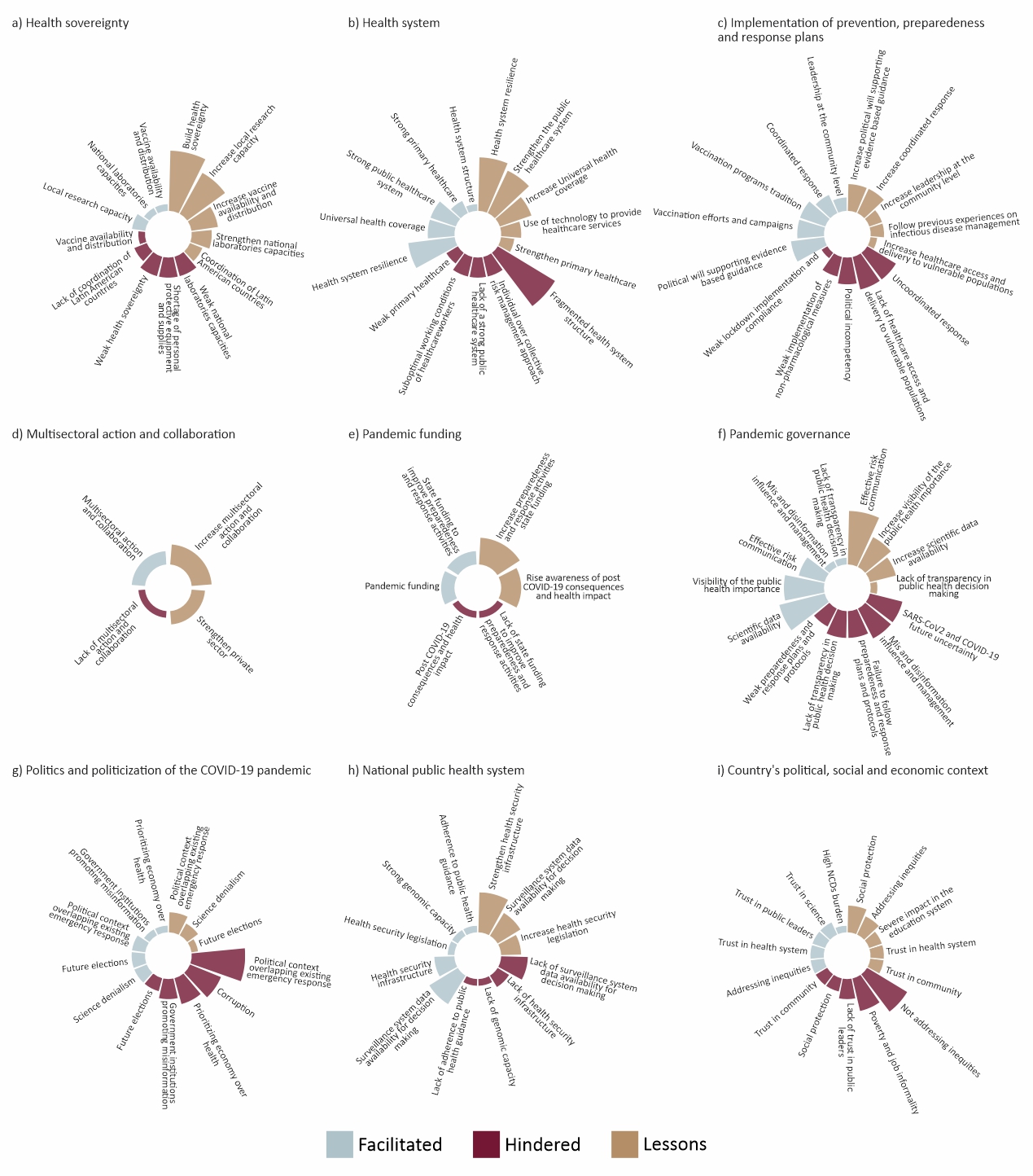


**Supplementary Figure 1: Main Themes Related to Pandemic PPR (a) Health Sovereignty; (b) Health System; (c) Implementation of PPR Plans; (d) Multisectoral Action and Collaboration, (e) Pandemic Funding, (f) Pandemic Governance, (g) Politics and Politicization of the COVID-19 Pandemic, (h) National Public Health System, and (i) Country’s Political, Social, and Economic Context for Pandemic Preparedness and Response During 2020–2021.**

# S8. Overall Perception of Support by National Decision Makers for Epidemic and Pandemic Preparedness and Response in the next 12 months.

**
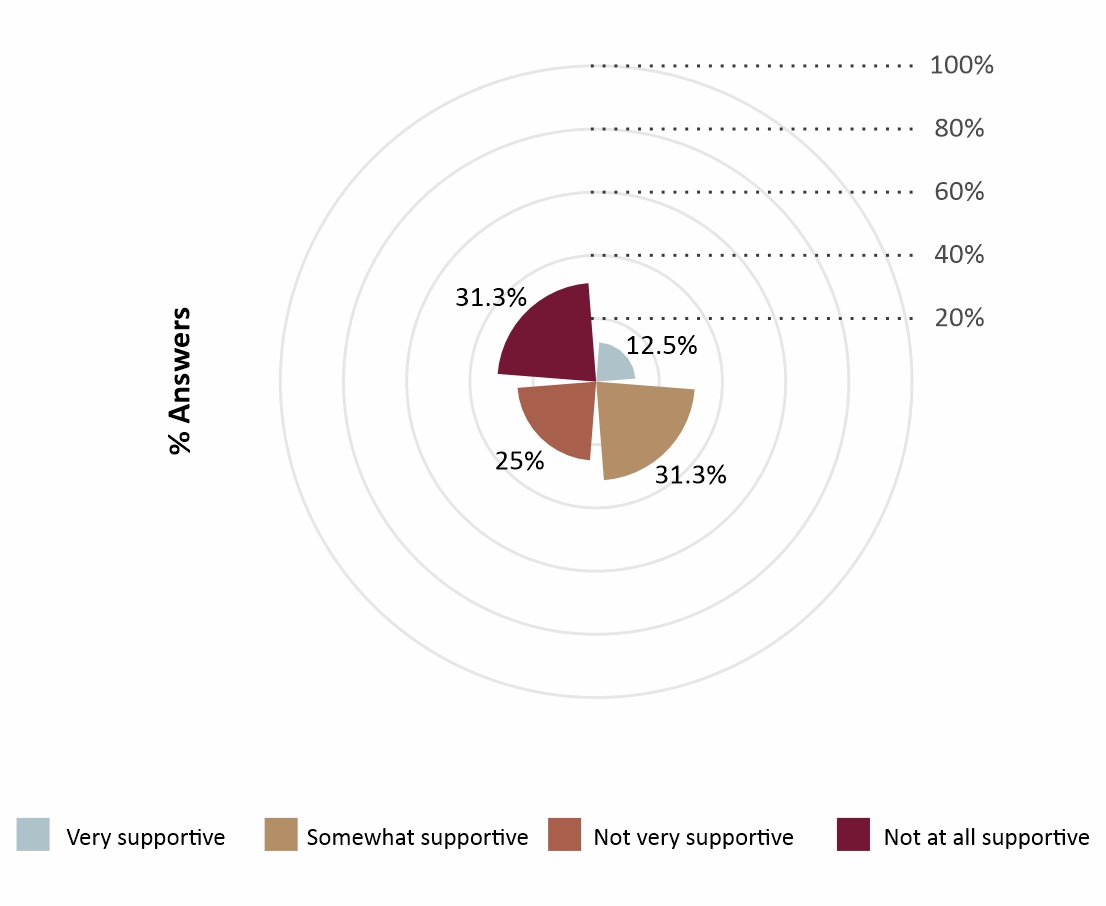
**

**Supplementary Figure 2: Overall Perception of Support by National Decision Makers for Epidemic and Pandemic Preparedness and Response in the next 12 months.**

# S9. Perception of national decision makers’ support for epidemic and pandemic Preparedness and Response in the next 12 months, by Country.


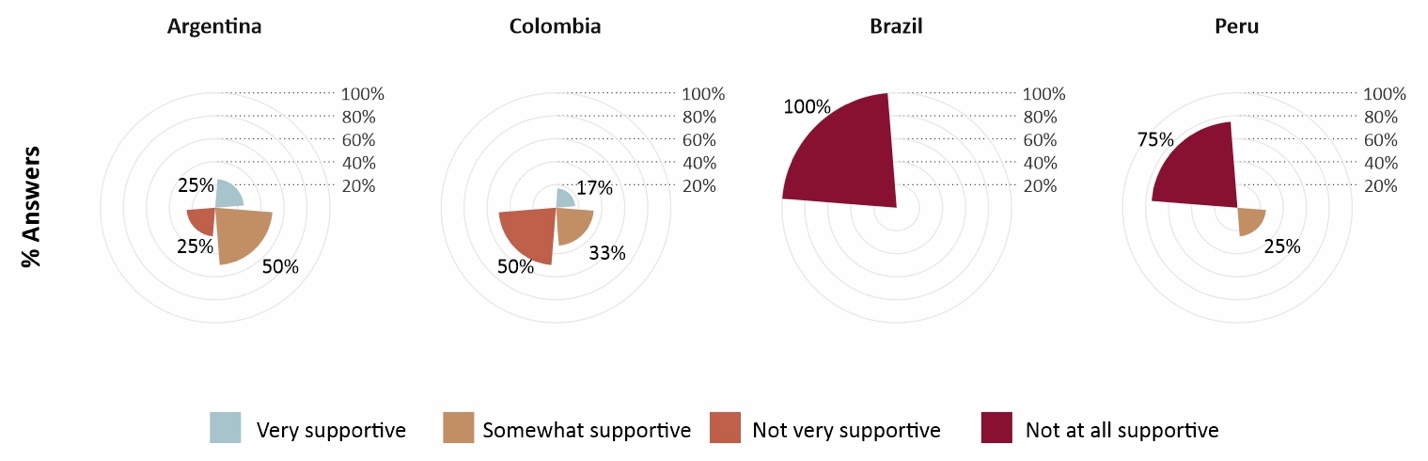


**Supplementary Figure 3: Perception of** n**ational** d**ecision** m**akers’ support for** e**pidemic and** p**andemic Preparedness and Response in the next 12 months, by Country.**

# S10. Perception of Top Key/Credible Organizations for Pandemic Prevention, Preparedness and Response Decision Making by Country.

| **Argentina** | **Brazil** | **Colombia** | **Peru** |
| --- | --- | --- | --- |
| Mayor’s Office / Municipality | Brazilian Health Regulatory Agency - ANVISA | Academia | Ombudsman’s Office |
| Medical associations | National Council of Health Secretaries - CONASS | Colombian Association of Infectious Diseases - ACIN | National Institute of Health - INS |
| National Scientific and Technical Research Council - CONICET | Butantan Institute | National Business Association of Colombia - ANDI | Round Table for the Fight against Poverty |
| Ministry of Social Development | Fiocruz Brasília | Regional authorities, mayor’s office, governor’s office, etc. | Minister of Economy and Finance |
| Municipal, provincial and national health teams | Ministry of Health | National Institute of Health - INS | Inter-governmental organisms |
| Ministry of Health / Disaster Division | Parliament | National Institute of Drug and Food Surveillance - INVIMA | Community-based organizations |
| Network of Laboratories, Vaccines, Therapeutics |  | Ministry of Health | Pan American Health Organization - OPS |
| Scientific societies |  | Pan American Health Organization - OPS | Presidency of the Council of Ministers - PCM |
| Universities / Academics |  | Health professionals | Peruvian radio programs |
|  |  | National Unit for Disaster Risk Management - UNGRD |  |

# S11. Perception of Top Areas/Priorities that Health Authorities and Policymakers will address in the next 12 months by Region and Country.

| **Argentina** | **Brazil** | **Colombia** | **Peru** | **Latin America** |
| --- | --- | --- | --- | --- |
| Preparedness for future pandemics | Depending on the electoral climate | Analysis and financial recovery of the implications of the pandemic for the country’s healthcare infrastructure | Healthcare reform | Consolidate the purchase of vaccines and supplies, tests, kits, etc. Price negotiation, corporate purchasing, having blocks of countries to do drug negotiations |
| Collaborate with vulnerable populations, social and economic programs | Investment in laboratory infrastructure | Pandemic management, development of new knowledge, concrete analysis for a national response plan, plan with these clear lessons learned | Health financing should be considered an investment for human development | COVID-related health infrastructure, education and children who have been harmed by the pandemic. Prioritization of investment in health infrastructure to ensure greater access and quality |
| Continue the vaccination plan | Integration of health records | Chronic disease reemergence, and post-COVID or long COVID syndrome | Health must be kept at the center of state policies | Human resources in health (meeting points, joint studies, transdisciplinarity, not only hospital doctors, prepared for the different realities, inclusive and integral health systems) |
| Expansion of the health system, modernization of hospitals infrastructure and laboratories, and re-evaluation of human resources | Translational academic research | Commitment of GDP to health in Colombia, remuneration, representativeness of the sector and personnel, need for other technical profiles in certain health areas in the country | Managing neglected diseases, displaced in diagnosis and treatment | Long COVID-19, better research, better diagnostics, better data, more robust studies, better case definitions |
| Psychological impact of the pandemic |  | Deterioration of public health indicators (mental health, domestic and gender violence) | Operational and logistical aspects to guarantee universal health care | Strengthening of genomic studies in the region, improved monitoring |
|  |  | Implementation of the single national health information system | Preparing for the next pandemic, long-term approach | Surveillance of COVID-19 disease and other COVID-19 related diseases/ syndemics |
|  |  | Production of supplies such as vaccines, critical patient/respiratory supplies and surveillance, diagnostic kits to support surveillance | Strengthening health systems, so that they have the capacity to respond to future pandemics or emergencies | Telemedicine for middle-income countries in particular |
|  |  |  | Strengthening the vaccination tradition | Professionalization of the interface between science and policy, determine gold standards - models - look at successful examples |
